# Chronic Exposure to 4-Nonylphenol Alters UDP-Glycosyltransferase and Sulfotransferase Clearance of Steroids in the Hard Coral, *Pocillopora damicornis*

**DOI:** 10.3389/fphys.2021.608056

**Published:** 2021-02-17

**Authors:** Luc R. A. Rougée, Abby C. Collier, Robert H. Richmond

**Affiliations:** ^1^Department of Tropical Medicine, Medical Microbiology and Pharmacology, John A. Burns School of Medicine, University of Hawaii, Honolulu, HI, United States; ^2^Kewalo Marine Laboratory, University of Hawaii at Manoa, Honolulu, HI, United States; ^3^Faculty of Pharmaceutical Sciences, The University of British Columbia, Vancouver, BC, Canada

**Keywords:** UDP-glycosyltransferase, steroids, coral, endocrine disruption, 4-nonylphenol, coral reproduction

## Abstract

The effects of the xenoestrogen 4-nonylphenol (4NP) on endocrine and metabolic homeostasis in the reef building coral, *Pocillopora damicornis* were investigated. The aim was to understand if ubiquitous nonylphenol ethoxylate contaminants in the marine environment result in altered homeostatic function. Coral colonies were chronically exposed (6 weeks) to a sublethal concentration (1 ppb) of 4NP and sampled over the coral’s lunar reproductive cycle. Although activity of steroidogenic enzymes [cytochrome P450 (CYP) 17, CYP 19, and 3-β-Hydroxysteroid dehydrogenase] and the conjugation enzyme glutathione-S-transferase was not altered, significant increases in the activity of the steroid clearing enzyme UDP-glycosyltransferase (UGT) were observed. The natural fluctuation of UGT activity with the lunar cycle was replaced with consistently high UGT activity throughout the reproductive cycle during 4NP exposure. No effect of 4NP on the reverse reaction, mediated by β-glucuronidase, was observed. Thus, 4NP shifts the UGT:β-glucuronidase ratio toward greater clearance at points in the lunar cycle where retention of compounds is typically favored. Additionally, 4NP reduced activity of the steroid regeneration enzyme steroid sulfatase, further shifting the system toward clearance rather than regeneration. These data imply that environmentally relevant levels of 4NP may be impacting the reproductive health of corals and threatening the persistence of coral reefs.

## Introduction

Both marine and terrestrial ecosystems are in decline from anthropogenic sources of stress. Specifically, for corals, over 30% of the world’s coral reefs have been lost in the last few decades, with an additional 30–40% at risk of being lost by the year 2050 (National Academies of Sciences, Engineering, and Medicine, 2019). These losses are due to both abiotic and biotic factors, including rapid increases in the rate and magnitude of climate change, as well as increases in the concentration and distribution of environmental contaminants affecting water quality on a global scale. Within the broad range of environmental contaminants, endocrine disruptors (EDs) have received a great deal of interest. Broadly defined, EDs are compounds that interfere with the production, release, transport, metabolism, binding, action, or elimination of natural hormones responsible for homeostasis and developmental processes (Kavlock et al., 1996). While initial concerns of endocrine disruption were focused on humans, the ability EDs to impact wildlife, especially marine invertebrates, was quickly realized (Tyler et al., 1998; Ford and LeBlanc, 2020). Early studies focused on the ability of compounds to bind to steroid receptors as a surrogate for endocrine disrupting effects. However, later discoveries such as the activation of the retinoid X receptor being responsible for the imposex observed in dog whelk (*Nucella lapillus*) exposed to tributylin, have demonstrated the complexity of mechanisms that can result in homeostatic disruption (Blaber, 1970; Castro et al., 2007).

The weak xenoestrogen, 4-nonylphenol (4NP) has garnered interest due to its wide use in consumer, pharmaceutical, and industrial products as a surfactant to increase miscibility of chemicals in compound mixtures (Ying et al., 2002). Although 4NP is present in consumer products in the ethoxylate form, the parent molecule re-emerges in the environment as a breakdown product (Ahel et al., 1994). The presence of 4NP has been measured globally in sewage treatment effluents, freshwater and marine ecosystems, and sediments (Soares et al., 2008). 4NP can persist in aquatic habitats, with an estimated half-life of 58 days (Ekelund et al., 1993), readily bind to organic matter (John et al., 2000; Langford and Lester, 2002), and remain in sediments with an estimated half-life greater than 60 years (Shang et al., 1999).

The toxicity of 4NP has been extensively studied in aquatic algae, invertebrates, and fish (Servos, 1999; Staples et al., 2004). In aquatic organisms, the effect of 4NP is frequently associated with estrogen receptor agonism, resulting in endocrine disruption (White et al., 1994; Giesy et al., 2000). The effects of 4NP in marine species have been less studied than for freshwater organisms. In the phylum Cnidaria, only the acute (96 h) toxic threshold of 4NP in *Hydra attenuata* (a freshwater invertebrate) has been investigated (Pachura et al., 2005; Pachura-Bouchet et al., 2006). While acute toxicities are important, there exists a disconnect between the high lethal concentrations determined in acute studies and the relatively low levels observed in the environment. In the marine aquatic environment, the range of 4NP concentrations range between 0.1 and 4 μg/L (ppb), with highest reported levels at 10 μg/L or greater in industrial areas (Bolz et al., 2001; Kawahata et al., 2004; Soares et al., 2008). Therefore, studies are needed to determine the effect of sublethal, chronic exposure to 4NP at environmentally relevant levels on key groups of organisms.

The current investigation was designed to evaluate the impact of chronic (6 weeks), low dose exposure to 4NP (1 ppb) on the reef building coral, *P. damicornis* over its monthly reproductive cycle. This coral was chosen as it produces brooded planula larvae on a monthly cycle throughout the year in Hawaii (Richmond and Jokiel, 1984). A comprehensive analysis of the impact of 4NP on the molecular endocrinology of *P. damicornis* is presented that includes an evaluation of 4NP effects on the levels of steroid hormones present in the coral tissue, the activities of steroidogenic enzymes, activities of steroid clearance/detoxification enzymes, and activities of steroid regeneration enzymes.

## Materials and Methods

### Chemicals

The following chemicals were used in this study: 4-methyl umbelliferone sodium salt (4-MU), MP Biomedicals (Solon, OH, United States); 4-methyl umbelliferyl-beta-D-glucuronide (4-MU-G), Acros Organics (Geel, Belgium); 3'-phosphoadenosine 5'-phosphosulfate (PAPS), Calbiochem (San Diego, CA, United States); NAD^+^ and NADPH, Calbiochem (San Diego, CA, United States); potassium hydroxide (KOH), Fisher Chemicals (Fair Lawn, NJ, United States); and pregnenolone, TCI America (Portland, OR, United States). All other chemicals were purchased from Sigma Aldrich Company (St Louis, MO, United States) and were analytical grade or higher.

### Coral Collection and Experimental Procedures

Whole coral colonies (~15 cm diameter; *n* = 6) of *P. damicornis* were collected from Coconut Island in Kaneohe Bay, Oahu under Special Activities Permit 2009-42, granted by the Hawaii Department of Land and Natural Resources, Division of Aquatic Resources. Corals were selected at random, cleaned of any foreign organisms and placed in a quarantine tank for 14 days prior to introduction into flowing seawater tanks at the Kewalo Marine Laboratory (KML). The quarantine tank consisted of an open flow through system sea water table. All water leaving the isolation tank was filtered and sterilized using a SMART Ultraviolet Sterilizer Model 02025 (Emperor Aquatics Inc., Pottstown, PA, United States) prior to release. After isolation, colonies were maintained in separate seawater tanks and allowed to recuperate for at least 28 days from collection-associated stress and allowed to acclimatize to the KML seawater system [salinity of 36 ± 1 parts per thousand (ppt), temperature 27 ± 2°C] prior to exposure. The Kewalo seawater system consisted of an unfiltered open flow through system where seawater was furnished through a pipe with an intake 300 m offshore in 10 m deep water.

### 4-Nonylphenol Solutions

Seawater used in the experiments was collected from the KML water system and filtered using 0.2 μm nitrocellulose membranes. A stock solution of 4NP was prepared in acetone at 0.1 mg/ml or 100 parts per million (ppm). Working solutions at the final concentration of 1 ng/ml or 1 part per billion (ppb) were prepared by the addition of 10 μl of the acetone stock to 1 L of filtered seawater. Final solutions were made fresh before each water change. Acetone was used a carrier solvent to dissolve 4NP into solution. The carrier solvent control stock solutions were prepared in the same manner as the 4NP solutions; with the exception that acetone alone was added.

### Coral Exposure to 4-Nonylphenol

Coral colonies were assigned into either the carrier solvent control (acetone) group (*n* = 3 whole coral colonies) or the 1 ppb 4NP group (*n* = 3 whole coral colonies). Dosing chambers for the exposure consisted of 2 L glass bowls, aerated using glass Pasteur pipettes and covered with Parafilm® to prevent evaporation. Dosing chambers were placed in water tables with running seawater to maintain temperature stability and were covered with a 50% shade cloth to decrease peak light levels. Temperature within the dosing chambers ranged from 25.5 to 28.5°C. Water changes were performed every 24–48 h.

Coral colonies were exposed to 4NP for a total of 6 weeks (42 days). No samples were taken for the first 2 weeks of exposure. After the initial 2 weeks, the corals were sampled on the lunar quarters (between 6 and 8 days), starting with the reproductive planulation event (0 time point), and ending the week before the following planulation event (−1 time point). Coral branches (~7 cm tall and 2.5 cm wide) were removed from the colony at the base of the coral where the skeleton was devoid of tissue to avoid further stress to the colony. Samples were placed in conical 50 ml polypropylene Falcon tubes, flash frozen with liquid nitrogen, and immediately placed in a −80°C freezer until further processing.

### Preparation of Whole Cell Lysates and S9 Subcellular Fractions From Coral

Corals tissue was removed from the skeleton using a Water Pik system and 0.2 μm filtered seawater (FSW; Johannes and Wiebe, 1970). The resulting coral blastate was transferred to conical 50 ml polypropylene Falcon tubes and spun at 10,000 *g* for 10 min at 4°C in a Sorvall RC-5B centrifuge (DuPont Instruments, San Pedro, CA, United States) to pelletize tissue and free-floating cells. The resulting pellets were resuspended and combined in 5 ml of cold homogenization buffer [FSW; 1 mM phenylmethylsulfonylfluoride (PMSF)] and homogenized on ice with an Ultra-Turrax homogenizer for 60 s. The homogenate was placed in a 15 ml Falcon tube and centrifuged at 2,000 *g* for 5 min at 4°C in an Eppendorf Centrifuge 5810R (Eppendorf, Hauppauge, NY, United States). The zooxanthellae pellet was discarded, and the supernatant was transferred to a new 15 ml Falcon tube and spun again at 2,000 *g* for 3 min to remove any remaining zooxanthellae. The final supernatant, representing whole cell lysate (WCL), containing only coral tissue, was aliquoted and frozen at −80°C until use. The S9 post-mitochondrial fraction of protein was obtained by processing the WCL using a glass homogenizer for 2 min (ca. 30 strokes). The resulting homogenate was centrifuged at 10,000 *g* for 10 min at 4°C in an Eppendorf 5415D Microcentrifuge (Eppendorf, Hauppauge, NY, United States). After this final centrifugation, the supernatant represents the S9 tissue fraction of coral. Protein concentrations of all samples were measured using the BCA method described by Smith et al. (1985) using bovine serum albumin as the protein standard.

### Quantifying Steroid Hormones

Measurements of cholesterol and steroidal hormones were performed on the coral tissue WCL. All WCLs were normalized to 0.25 mg/ml for total and free cholesterol and 2 mg/ml for steroid hormone ELISA assays. Total and free cholesterol were measured by biochemical assay as per the manufacturer’s instructions (Cayman Chemical, Ann Arbor, MI, United States). The steroid hormones estrone, 17β-estradiol, testosterone, and progesterone were measured by ELISA (ALPCO Immunoassays, Salem, NH, United States; Calbiotech, Spring Valley, CA, United States for progesterone), per the manufacturer’s instructions. The specificity of antibodies raised to chemical structures is high, with cross reactivity to structurally related compounds being less than 6% for cholesterol, estrone, 17β-estradiol, testosterone, and progesterone, respectively.

### Enzyme Assays

All steroidogenic assays were performed in 5 ml glass tubes, unless specified, and subsequently aliquoted in triplicate into 96-well clear microplates. For clearance and regeneration enzyme assays, coral protein, assay buffer, and substrate were loaded into wells on microplates that were kept on ice. Microplates were pre-warmed inside a microplate reader (Spectra Max or Gemini XS, Molecular Devices, Sunnyvale, CA, United States) at 37°C for 5 min prior to the addition of the co-factors used to initiate the reaction. Fluorescence assays were performed in black, flat-bottomed plates, colorimetric assays in clear plates, and UV assays in optically clear microplates. For substrates dissolved in solvents, solvent was never more than 2% of the reaction volume; hence, solvent carrier properties should not have affected enzyme activities (Williams et al., 2008). Microplates were read using either a Spectra Max 340 Plus or Gemini XS (Molecular Devices, Sunnyvale, CA, United States). Individual enzyme activities were assessed as follows:

#### 3-β-Hydroxysteroid Dehydrogenase

The activity of 3-β-Hydroxysteroid dehydrogenase (3βHSD) was determined by measuring the conversion of pregnenolone to progesterone. Glass tubes were kept on ice while coral WCLs (0.2 mg/ml final concentration) in 0.1 M Tris-HCl buffer pH 7.4 containing 50 mM MgCl_2_ and 150 ng/ml pregnenolone were added. Tubes were pre-incubated for 30 s at 37°C in a hot water bath. Reactions were initiated through the addition of 1 mM NAD^+^ and incubated at 37°C for 20 min. After incubation, the reaction was terminated by plunging tubes into ice for 5 min. Detection of progesterone was determined through ELISA and converted to ng/min/mg of protein using a standard curve of progesterone (manufacturer supplied).

#### Cytochrome P450 17/17 α-Hydroxylase

Activity of CYP17 was determined using a method adapted from Holtroff and Koch (1940). Briefly, coral protein (0.2 mg/ml final concentration) in 0.1 M Tris-HCl buffer pH 7.4 containing 50 mM MgCl_2_ and 500 μM 17α-hydroxypregnenolone were added to glass tubes on ice. Tubes were pre-incubated for 30 s at 37°C prior to reaction initiation through the addition of 1 mM NADPH. Tubes were then incubated at 37°C for 5 min. Reactions were terminated through the addition of an equal volume of 5 M KOH. An equal volume of 2% m-dinitrobenze, in 95% ethanol, was added and the color allowed to develop for 2 min. The mixture was transferred to 1.5 ml microcentrifuge tubes and spun at 10,000 *g* for 3 min in an Eppendorf Minispin centrifuge (Hauppauge, NY, United States) to pelletize the precipitate. Absorbance was determined at *λ* = 520 nm in triplicate wells and results transformed to nmoles/min/mg of protein using a standard curve generate through dilution of pure DHEA (0–1 mM).

#### Cytochrome P450 19/Aromatase

The activity of CYP19 was determined by measuring the conversion of testosterone to 17β-estradiol. Briefly, glass tubes were kept on ice while coral WCLs (0.2 mg/ml final concentration) in 0.1 M Tris-HCl buffer pH 7.4, containing 50 mM MgCl_2_, and 10 ng/ml of testosterone were added. Tubes were pre-incubated at 37°C in a hot water bath. Reactions were initiated through the addition of 1 mM NADPH and the tubes incubated at 37°C for 30 min. After incubation, the reaction was terminated by placing the tubes into ice for 5 min. Detection of estradiol was determined through ELISA and data were converted to fg/min/mg of protein using a standard curve of progesterone (manufacturer supplied).

#### Glutathione-S-Transferase

Activity of glutathione-S-transferase (GST) was determined using a method adapted from Habig et al. (1974) and González et al. (1989). Optically clear microplates (Greiner Bio-One, Monroe, NC, United States) were placed on ice and loaded with 0.5 mM 1-chloro-2,4-dinitrobenzene (in DMSO) and coral protein (0.1 mg/ml). After pre-incubation (3 min at 37°C), reactions were initiated through the addition of 1 mM L-glutathione. Absorbance was monitored continuously at *λ* = 340 nm. Total activity was calculated using Beer’s Law with *ε* = 9.6 mM^−1^ cm^−1^ (Habig et al., 1974).

#### UDP-Glycosyltransferase

Total UDP-glycosyltransferase (UGT) activity in coral protein (0.3 mg/ml) was determined using the method of Collier et al. (2000) with substrate 4-methylumbelliferone (4MU). The method was modified so that 50 μg/ml alamethicin (in DMSO) was used as the UGT activator and 5 mM sacchrolactone was included in each reaction to inhibit β-glucuronidase activity. Fluorescence was monitored continuously at 355 nm ex/460 nm em and results were transformed to pmol/min/mg protein using a standard curve generated with 4-MU.

#### β-Glucuronidase

β-Glucuronidase activity was determined using the method of Trubetskoy and Shaw (1999). Microplates on ice were loaded with coral protein (0.1 mg/ml final concentration) and buffer; plates were pre-incubated at 37°C for 5 min and the reaction initiated with 100 μM 4MU-Glucuronide. Fluorescence was continuously monitored at 355 nm ex/460 nm em. Results were transformed to pmol/min/mg protein using a standard curve generated with 4-MU.

#### Sulfotransferase 1A1

The activity of sulfotransferase 1A1 (SULT1A1) was measured using the method of Frame et al. (2000). Coral protein (1 mg/ml final concentration), 5 mM para-nitrophenyl sulfate, and 0.1 mM 2-naphthol were added to each well of a microtiter plate, pre-incubated for 5 min at 37°C, and then the enzyme reaction was initiated by the addition of 60 μM 3'-phosphoadenosine 5'-phosphosulfate (PAPS). Reactions proceeded for 1 h, and then absorbance was measured at *λ* = 405 nm. Total SULT1A1 activity was calculated using Beer’s Law with *ε* = 18.2 mM^−1^ cm^−1^ (Frame et al., 2000).

#### Steroid Sulfatase

Activity of the arylsulfatase isoform C, referred to as steroid sulfatase (STS), was determined using a modification of the method of Roy (1958). Microplates were kept on ice and coral protein in 0.1 M Tris-HCl buffer pH 7.4 containing (0.5 mg/ml final concentration) was added to each well. The samples were pre-incubated for 5 min at 37°C, and then reactions were initiated with the addition of 100 μM para-nitrophenyl sulfate. Absorbance was monitored continuously at *λ* = 400 nm and results were generated using a standard curve of para-nitrophenol.

### Statistical Analyses

Statistical analyses were performed using Prism 8.0 with statistical significance set at *α* = 0.05. (GraphPad Prism, San Diego CA, United States). Parametric statistics were performed, since all data approximated Gaussian distributions, and two-tailed student *t*-tests were used to assess differences between groups, with an *F*-test to compare variances. When paired sample *t*-tests were performed, the correlation coefficient was generated to assess the effectiveness of the pairing.

## Results

### Steroid Hormones

**Figure 1:**
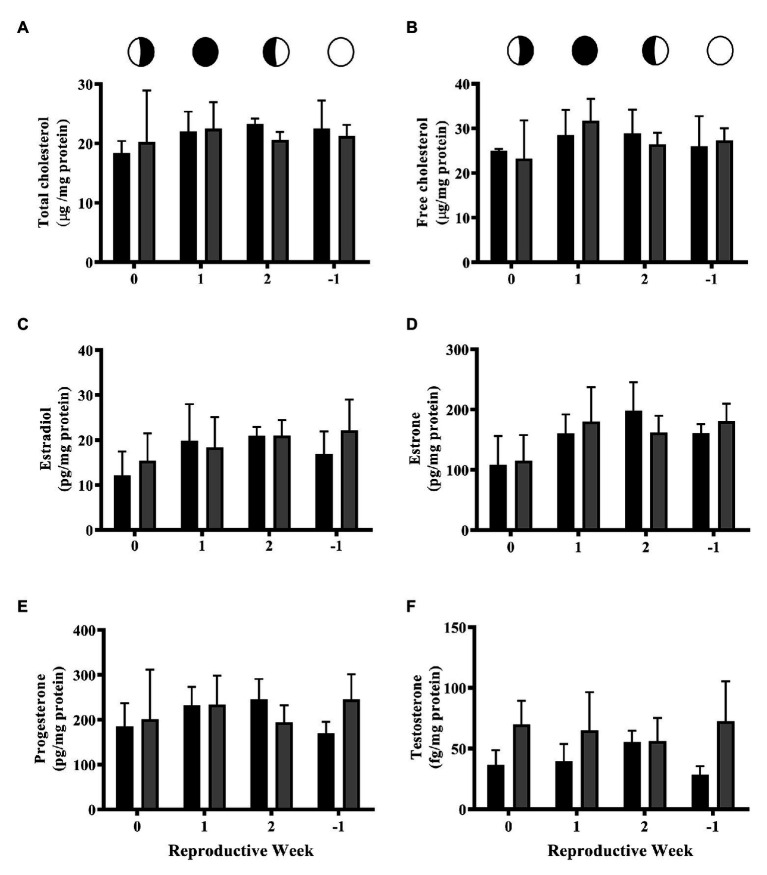
Levels of cholesterol and steroid hormones over the lunar reproductive cycle in control corals and corals exposed to 4NP. **(A)** Total cholesterol, **(B)** free cholesterol, **(C)** 17β-estradiol, **(D)** estrone, **(E)** progesterone, and **(F)** testosterone. Circles with different fills represent the lunar cycle period in the order of 3rd quarter moon (planulation, time point 0), new moon (1 week after planulation, time point 1), 1st quarter moon (2 weeks after planulation, time point 2), and full moon (1 week before planulation, time point −1). Solid black bars represent control corals and gray bars represent corals chronically expose to 4NP. Bars represent mean ± SD for *n* = 3 experiments, each assayed in triplicate.

No significant differences were detected between controls (CTL) and 4NP exposed corals at any time point in the lunar cycle for total or free cholesterol (Figures 1A,B). The same trend was observed for all steroid hormones (Figures 1C–F). A trend of increased testosterone in the 4NP exposed corals at the 0 and −1 time points, respectively, (*p* = 0.0839 and 0.0608, respectively, *t*-test), was observed, although these did not reach statistical significance (Figure 1F).

### Steroidogenic Enzymes

**Figure 2:**
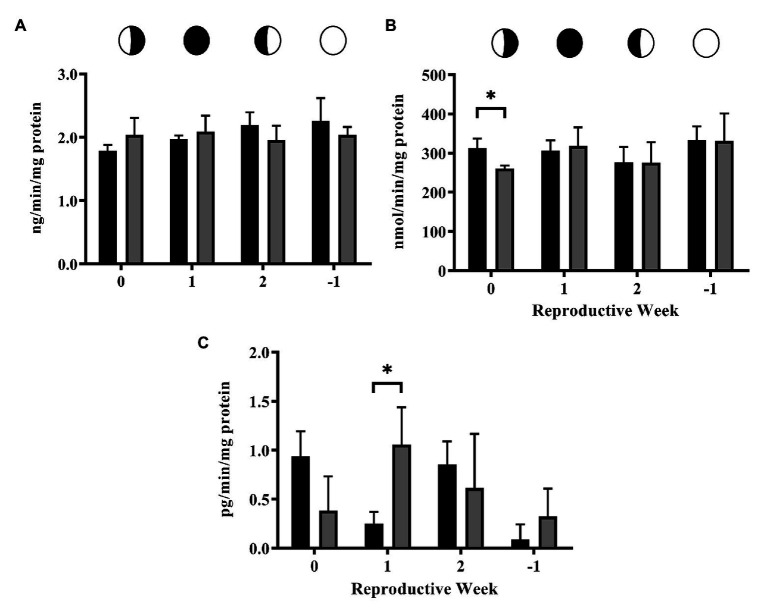
Activity of steroidogenic enzymes over the lunar reproductive cycle in control corals and corals exposed to 4NP. **(A)** 3βHSD, **(B)** CYP17, and **(C)** CYP19. Circles with different fills represent the lunar cycle period in the order of 3rd quarter moon (planulation, time point 0), new moon (1 week after planulation, time point 1), 1st quarter moon (2 weeks after planulation, time point 2), and full moon (1 week before planulation, time point −1). Solid black bars represent control corals and gray bars represent corals chronically expose to 4NP. ^*^p ≤ 0.05 (unpaired *t*-test). Bars represents mean ± SD for *n* = 3 experiments, each assayed in triplicate.

Activity of 3βHSD was not altered by 4NP (Figure 2A). However, some significant effects on the activity of CYP17 and CYP19 were caused by 4NP exposure. The activity of CYP17 was significantly lowered by exposure to 4NP (*p* = 0.027) at the 0 time point (Figure 2B), while CYP19 activity was significantly higher as a result of 4NP exposure (*p* = 0.025) at the 1 time point (Figure 2C). Additionally, CYP19 activity of the 4NP exposed corals remained consistent over the lunar cycle with none of the levels at the different time points being significantly different from one another (Figure 2C). This is in contrast to the control corals whose CYP19 activity was significantly lower at the 1 and −1 time points compared to the other time points in the lunar cycle (Figure 2C).

### Steroid Conjugation/Deconjugation Enzymes

**Figure 3:**
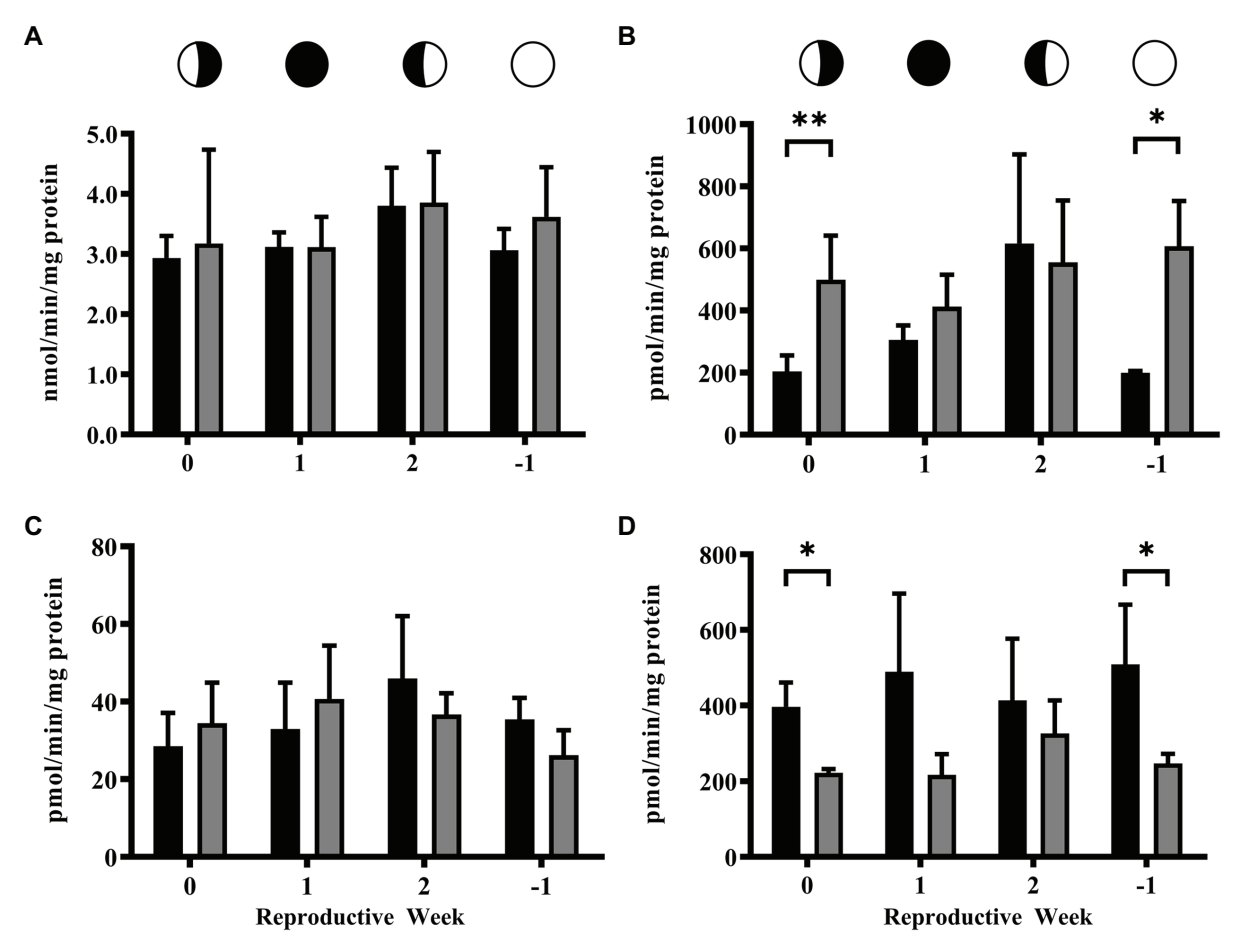
Activity of steroid clearance/detoxification and regeneration enzymes over the lunar reproductive cycle in control corals and corals exposed to 4NP. **(A)** GST, **(B)** UGT, **(C)** β-glucuronidase, and **(D)** STS. Circles with different fills represent the lunar cycle period in the order of 3rd quarter moon (planulation, time point 0), new moon (1 week after planulation, time point 1), 1st quarter moon (2 weeks after planulation, time point 2), and full moon (1 week before planulation, time point −1). Solid black bars represent control corals and gray bars represent corals chronically expose to 4NP. Bars are mean ± SD for *n* = 3 experiments, each assayed in triplicate. ^*^p ≤ 0.05 and ^**^p ≤ 0.01 (unpaired *t*-test).

Activity of the conjugation enzyme GST did not fluctuate over the lunar cycle and was unaffected by exposure to 4NP (Figure 3A). In contrast, the activity of UGT enzymes for the CTL corals fluctuated over the lunar cycle, as previously reported (Rougée et al., 2014, 2015), with significantly higher UGT activity at the 2 time point compared to the 0 and −1 time points (One-way ANOVA *p* = 0.026; Tukey’s Multiple Comparison Test, Figure 3B). However, UGT activity in 4NP exposed corals did not fluctuate over the lunar cycle and was significantly higher compared to that of CTL corals at the 0 and −1 time points (*p* = 0.027 and 0.009, respectively; two tailed unpaired *t*-test). The regeneration enzyme, β-glucuronidase, activities did not fluctuate over the lunar cycle and were not altered in the presence of 4NP (Figure 3C). The increase in UGT activity on the 0 and −1 time points as a result of 4NP exposure, coupled with a lack of change in β-glucuronidase, resulted in an increase of UGT:β-glucuronidase activity ratio of 2- and ~4-fold, respectively, for the 0 and −1 time points (Table 1). This shifted the balance toward elimination as opposed to retention at the given time points. The activity of the reverse clearance enzyme STS was reduced over the lunar reproductive cycle. This reached significance at the 0 and −1 time points (*p* = 0.009 and 0.047, respectively; two tailed unpaired *t*-test; Figure 3D).

**Table 1:** Changes in the UGT:β-glucuronidase enzyme ratio over the coral lunar reproductive cycle time points.

	Lunar time point			
Condition	0	1	2	−1
Control	7:1	9:1	13:1	6:1
4-nonylphenol exposed	15:1	10:1	15:1	23:1
Fold change	2.1	1.1	1.2	3.8

0, planulation (3rd quarter moon); 1, 1 week after planulation (new moon); 2, 2 week after planulation (1st quarter moon); −1, 1 week before planulation (full moon).

Finally, the activity of SULT1A1 was not detected. The carrier solvents acetone and methanol have been found to significantly reduce and inhibit the activity of sulfotransferases (Arand et al., 1987; Ma et al., 2003). Since acetone exposure occurred over a prolonged period (6 weeks), a 1% final concentration of methanol was used as a carrier solvent for PMSF in the homogenization buffer, and SULT1A1 activity could not be detected in either the control or exposed coral, it is suspected that SULT1A1 activity was significantly affected by the presence of these carrier solvents in the experimental design.

### Planulae Release

**Figure 4:**
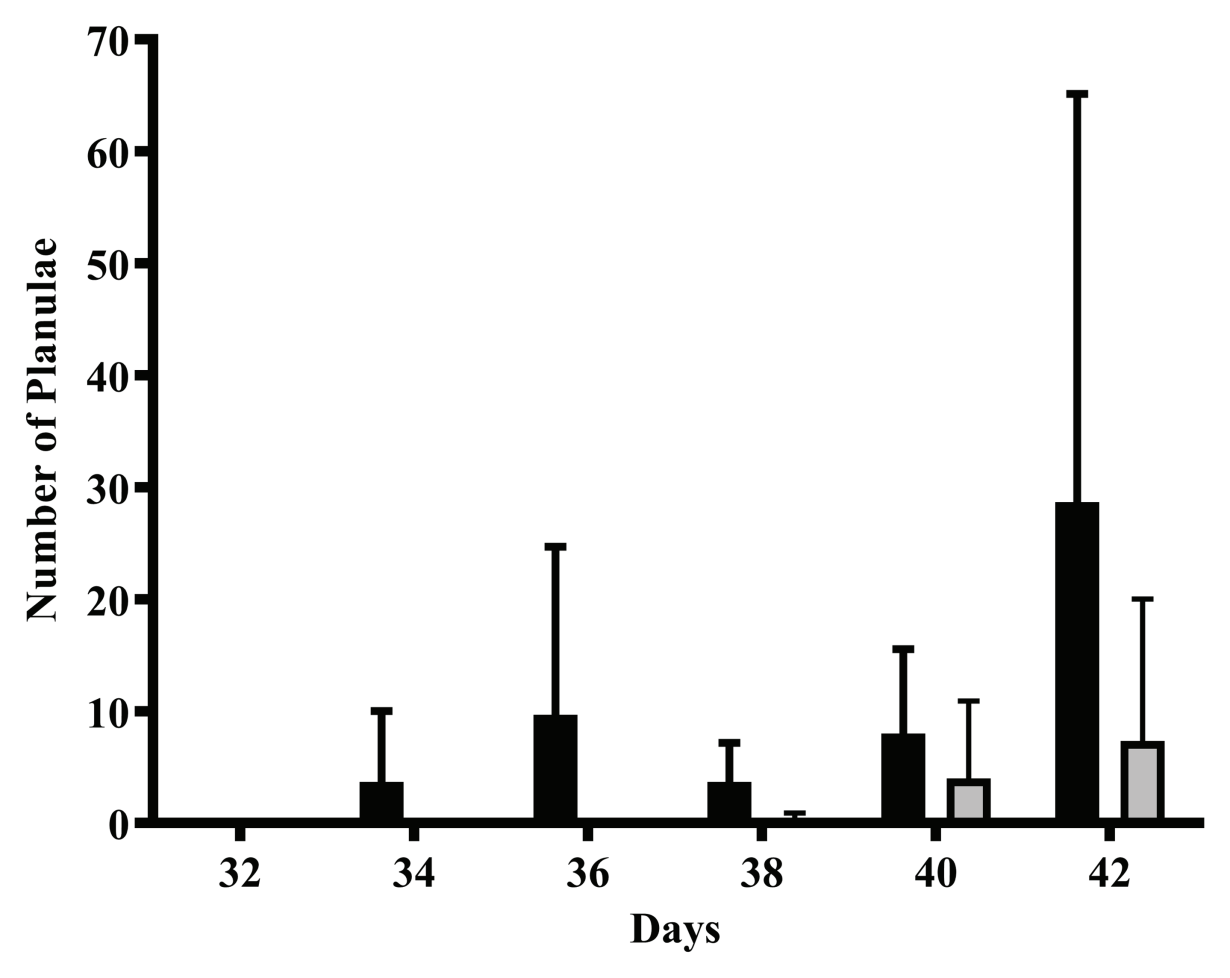
The effect of 4NP exposure on the release of planulae leading up to the subsequent planulation event. Solid black bars represent control corals and gray bars represent corals chronically expose to 4NP. Error bars are mean ± SD for *n* = 3 separate experiments (in triplicate each time).

Planula larvae released during the first 4 weeks of the exposure was low to non-detectable. Planulae production began to increase in the weeks approaching the next planulation event (2 weeks before and 1 week before the lunar reproductive peak). A greater number of planulae were released by the CTL colonies compared to the 4NP exposed corals; however, this did not reach statistical significance due to the high variability between colonies (Figure 4).

## Discussion

Our data indicate that chronic environmental exposure to sublethal concentrations of 4NP results in changes in biotransformation and detoxification enzymes in the coral *P. damicornis*, affecting steroid homeostasis and endo- and xeno-biotic detoxification. Significant dysregulation was observed in enzymes associated with the detoxification of 4NP itself (UGT) as well as in the regulation of steroid hormones (UGT and STS; Müller et al., 1998), which may affect coral reproductive fitness and constrain dynamic protein protective responses to additional stressors such as reduced water quality and associated toxicant exposure.

Previous studies using the coral *P. damicornis* observed significant variation in activity of the UGT enzyme over its lunar reproductive cycle, with low activity the week before and the week of planulation, followed by increased activity peaking 2 weeks after/before the monthly planulation event. This suggests that UGT activity is associated with the formation of planulae (Rougée et al., 2014, 2015). In the current study, exposure to 4NP resulted in sustained increases in UGT activities, possibly associated with the dual role of UGT in detoxification. While many studies in invertebrates explore the activities of the CYP enzymes, less attention has been given to the role of conjugation enzymes in the detoxification of xenobiotics. Recent research has identified the presence of UGT enzymes in a variety of invertebrates, linking them to pesticide detoxification as well as providing a mechanism for pesticide resistance (Erban et al., 2017; Li et al., 2018; Pan et al., 2018; Zhou et al., 2019). In the case of 4NP, glucuronidation is a predominant pathway for elimination in a variety of organisms including rats, fish, and cockroaches (Knaak et al., 1966; Coldham et al., 1998; Daidoji et al., 2006). Therefore, it is likely that the observed increase in UGT in *P. damicornis* has a role in the metabolic elimination of 4NP from the system, in addition to other homeostatic roles such as steroid balance.

The effect of the change in UGT activity extends beyond detoxication and into pathways associated with reproduction. The activity of the reverse cleavage enzyme, β-glucuronidase, remained consistent over the lunar cycle and did not fluctuate in response to 4NP exposure. The ratio of these enzymes (UGT:β-glucuronidase) can provide insights into the balance of homeostasis by describing the predominant direction of metabolism: removal or regeneration of compounds. This ratio has previously been shown to shift over the reproductive cycle of *P. damicornis* with higher rates of clearance for endo- and xeno-biotics 2 weeks before and 2 weeks after planulation, as compared to the intervening weeks where the balance of UGT: β-glucuronidase entailed a greater component of recycling (Rougée et al., 2014, 2015). The dynamic changes in the UGT:β-glucuronidase ratio over the reproductive cycle were driven by changes in the UGT enzymes, not β-glucuronidase. In the current study, the increase in the ratio of UGT:β-glucuronidase throughout the reproductive lunar cycle for *P. damicornis* resulted in higher rates of clearance for compounds at points in the lunar cycle where conservation of chemicals is favored. Empirically, skewing the balance of chemical clearance/regeneration in this manner would cause excessive excretion of endogenous ligands (such as steroids) normally conserved for use in coral tissues during this time period. The release of steroid hormones from coral tissue into surrounding seawater has been observed as a cue for reproductive events in corals (Twan et al., 2006). Therefore, dysregulation in the timing and release of steroid hormones into the seawater could potentially result in altered reproductive events. Additionally, the constant presence of steroid hormones in the environment can decrease reproductive potential as was previously observed in the coral *Montipora capitata* when exposed to continuous estradiol levels for 3-weeks prior to a reproductive event (Tarrant et al., 2004).

One of the major metabolic routes of estradiol elimination is glucuronidation (Hum et al., 1999). Consequently, upon 4NP exposure, the estradiol tissue levels would be expected to decrease in response. However, our current study did not detect a decrease in estradiol. We ascribe this to an observed increase in CYP19 (aromatase) activity which is responsible for the conversion of C19 steroids (e.g., testosterone) into estradiol (Janer and Porte, 2007). Therefore, we propose a dynamic balance occurring where increases in CYP19 are in response to greater UGT activity as an adaptive response to environmental stimuli, to maintain endocrine homeostasis. While this was observed as a trend, the lack of statistical significance leads us to cautiously propose this mechanism. There is clear evidence that toxicants can impact alternate pathways affecting endocrine homeostasis. For example, it was recently demonstrated that chronic exposure of the bumblebee, *Bombus terrestris*, to the pesticide imidacloprid suppressed the mevalonate pathway, which is tightly connected to the synthesis of sterols (Goldstein and Brown, 1990; Erban et al., 2019). Affecting the synthesis of sterols is harmful for non-targets, and these effects can be chronic, affecting direct synthesis of steroid hormones and thus behavior and reproduction (Crisp et al., 1998; Kuervers et al., 2003; Clotfelter et al., 2004; Baglan et al., 2018).

In contrast to consistently elevated UGT activity during the reproductive cycle as a result of 4NP exposure, STS enzyme activity was consistently decreased. This affects another major conjugation pathway intimately associated with steroids, namely sulfation. Similar to the UGT: β-glucuronidase inter-relationship, regulation of steroid hormones and other endogenous compounds can be affected by the balance between SULT:STS clearance and regeneration partners (Mueller et al., 2015). As an example, the steroid hormone dehydroepiandrosterone (DHEA) is regulated by this pathway in most mammals (except rodents; van Weerden et al., 1992; Mueller et al., 2015). A circulating reservoir of the sulfonylated (at the oxygen atom) metabolite, dehydroepiandrosterone sulfate (DHEAS), is maintained until the need for DHEA arises (Mueller et al., 2015; Geyer et al., 2017; Gabai et al., 2020). When DHEA is required, STS enzymes cleave the sulfate to regenerate the parent compound. This is bio-energetically conservative for the organism as compared to *de novo* synthesis. Therefore, decreases in STS activity can impact this delicate balance, forcing the organism to compensate for decreased levels of recirculated enzyme through *de novo* production (Garbarino and Sturley, 2005; Tobler et al., 2007). Aside from mammalian physiology, it is known that STS is important for desulfonation of DHEAS in normal growth and development, as well as controlling the rate of deconjugation of estrone sulfate in many organisms, likely also of relevance to coral as well (Mueller et al., 2015). If the coral maintains a reservoir of a sulfonylated ligand (such as DHEAS or estrone-S) that requires cleavage by STS for regeneration into the active parent ligand (DHEA and estrone, respectively), inhibition of STS could alter the finely-tuned balance of these chemicals. Furthermore, levels of the parent compound will decrease as sulfonylation continues and, in the case of corals reported here, glucuronidation is increased. This would force corals to compensate by producing more parent compound *de novo*, which is both energetically costly for the organism and may be disruptive at local tissue levels (Brown and Sharpe, 2016). The opportunity cost of *de novo* synthesis is likely to affect all processes in which these molecules take part, including behavior, development, and reproduction. This is consistent with our observation that the corals exposed to 4NP released few planula during the days around the reproductive planulation event as compared to the control corals. The duality of protein functions in organisms is not surprising, but is also concerning. Allocation to detoxification over reproduction is adaptive for immediate survival of individuals, but deleterious to long-term survival at the population level, ultimately with serious consequences for the persistence and survival of corals reefs.

In contrast to the other major Phase II enzymes, GST activities in corals did not change with exposure to 4NP. Previous findings in the mussel *Mytilus galloprovincialis*, showed a dose-dependent increase in GST activities upon chronic exposure (30 days) to 4NP, suggesting upregulation of GST as a survival mechanism (Vidal-Liñán et al., 2015). Although we did not observe a change in GST in corals, this is likely a dose-response effect. GST activity was significantly increased at 50 μg/L (50 ppb) or greater in the mussel study cited above, which is exponentially higher than our current study (1 ppb). The observation by Vidal-Liñán et al. (2015) that the increased activity of GST in response to 4NP exposure continued to increase even after a 10-day depurination period is concerning as it suggests that 4NP accumulates in the tissues over chronic exposure. In addition to dose-response effects, inter-species and inter-strain differences within a species are expected. This is also illustrated by Riva et al. (2010), who observed no change in GST activity in the zebra mussel (*Dreissena polymorpha*) at 4NP concentrations up to and including 50 μg/L (ppb). Therefore, even without considering that coral is a very different invertebrate to bivalve mollusks, even with the species “mussel” at 50 μg/L, 4NP *M. galloprovincialis* has significantly deregulated GST, but *D. polymorpha* did not (Riva et al., 2010; Vidal-Liñán et al., 2015). This highlights the care needed when comparing dynamic responses between organisms. Although we did not observe changes at the lower concentration of 4NP, it is possible that given a longer exposure, significant accumulation of 4NP levels could be reached that would elicit an effect.

Future assessments of sublethal exposure to toxicants should balance the length of exposure and concentration in order to fully understand the chronic effects these compounds may have. This could help to determine causation for the initial significant decrease in CYP17 activity in 4NP exposure or the lack of activity changes in 3βHSD observed in this study, both upstream pathways to the formation of testosterone (Janer and Porte, 2007). Finally, it is important to stress that while many inferences of steroid biochemistry pathways are made from the extensive knowledge of human pathways, and despite the commonality of steroids in Animal Kingdom; their regulatory mechanisms almost certainly vary (Ford and LeBlanc, 2020).

The presence of an array of steroid production and elimination proteins in simple, evolutionarily primitive organisms such as corals is unsurprising, considering their success over millions of years, the fundamental role(s) of steroids in animals, and the multiplicity of overlapping roles for detoxication enzymes. In a world of increasing anthropogenic stressors, particularly widespread toxicants in the marine environment, the ability of diverse taxa of invertebrates to express protective proteins is essential to their survival. Indeed, xenobiotic metabolizing enzymes are under considerable evolutionary pressure and continue to evolve widely in many animal, insect, and plant species in response to increasing environmental exposure (Bock, 2016). The expression of conjugation enzymes, that play role(s) in homeostasis for nutrients, hormones, and signaling pathways, as well as detoxication is foundational to the survival of both individuals and ecosystems. Further investigation to identify other such enzymes, as well as fully characterize and understand the role of these proteins within corals and other invertebrates is critical for scientifically based interventions to understand the dynamic interplay between exposure and adaptation. Continued development of these types of tools, such as the ones presented herein, to help diagnose the effect of xenobiotic contaminants can improve efforts toward the protection and restoration of coral reefs that are of great ecological, economic, and cultural value world-wide.

## Data Availability Statement

The raw data supporting the conclusions of this article will be made available by the authors, without undue reservation.

## Author Contributions

LR, RR, and AC participated in research design, contributed to new reagents, or analytical tools, performed data analysis, and wrote or contributed to the writing of the manuscript. LR conducted experiments. All authors contributed to the article and approved the submitted version.

### Conflict of Interest

The authors declare that the research was conducted in the absence of any commercial or financial relationships that could be construed as a potential conflict of interest.
